# Citicoline and COVID-19-Related Cognitive and Other Neurologic Complications

**DOI:** 10.3390/brainsci12010059

**Published:** 2021-12-31

**Authors:** Yuda Turana, Michael Nathaniel, Robert Shen, Soegianto Ali, Rajender R. Aparasu

**Affiliations:** 1 School of Medicine and Health Sciences, Atma Jaya Catholic University of Indonesia, Jl. Pluit Raya No. 2, North Jakarta 14440, Jakarta, Indonesia; michaelnathanielb@gmail.com (M.N.); robertshen388@gmail.com (R.S.); soegianto.ali@atmajaya.ac.id (S.A.); 2 Department of Pharmaceutical Health Outcomes and Policy, College of Pharmacy, University of Houston, 4849 Calhoun Road, Room, Houston, TX 4052, USA; raparasu@CENTRAL.UH.EDU

**Keywords:** COVID-19, cognitive, citicoline, neurologic, treatment

## Abstract

With growing concerns about COVID-19’s hyperinflammatory condition and its potentially damaging impact on the neurovascular system, there is a need to consider potential treatment options for managing short- and long-term effects on neurological complications, especially cognitive function. While maintaining adequate structure and function of phospholipid in brain cells, citicoline, identical to the natural metabolite phospholipid phosphatidylcholine precursor, can contribute to a variety of neurological diseases and hypothetically toward post-COVID-19 cognitive effects. In this review, we comprehensively describe in detail the potential citicoline mechanisms as adjunctive therapy and prevention of COVID-19-related cognitive decline and other neurologic complications through citicoline properties of anti-inflammation, anti-viral, neuroprotection, neurorestorative, and acetylcholine neurotransmitter synthesis, and provide a recommendation for future clinical trials.

## 1. Introduction

The World Health Organization in March 2020 declared COVID-19 as the 5th pandemic in the world after the Influenza A H1N1 virus pandemic in 1918 (Spanish flu), H2N2 in 1957 (Asian flu), H3N2 in 1968 (Hong Kong flu), and H1N1 (Pandemic flu) in 2009 [1]. COVID-19 is caused by severe acute respiratory syndrome coronavirus (SARS-CoV-2) and is the first digitally documented pandemic since its commencement [1]. Initially discovered in December 2019 in Wuhan, China, COVID-19 rapidly became a global pandemic and the biggest acute pandemic catastrophe in human history [2,3,4]. The current rapid development of COVID-19 vaccines does not negate the need for more effective treatment options considering that healthcare facilities continue to be overwhelmed in many areas [5,6].

An early study from Wuhan reported that neurologic symptoms were observed in more than one-third (36.4%) of COVID-19 patients [7]. Moreover, a study by Miskowiak et al. revealed more than 80% of COVID-19 patients with prolonged symptoms in 3 to 4 months after discharge reported having severe cognitive impairment in daily life, which was associated with more subjective cognitive difficulties, absenteeism, and poorer quality of life [8]. Some groups of non-hospitalized COVID-19 patients are termed as “long haulers” since they develop relatively mild symptoms that are persistent 4.72 months to 5.82 months after symptom onset; the single most common symptom reported is fatigue, but 85% of them experience four or more neurologic symptoms, with the most frequent symptom being “brain fog” with worsening of attentional function and working memory, representing a mild form of post-COVID-19 encephalopathy [9,10]. Brain fog is a collective term for CNS involvements inclusive of cognitive impairments, poor concentration, confusion, psychological symptoms, and behavioral changes [11]. Neurological symptoms found among the “long haulers” (irrespective of their positive or negative PCR tests results for SARS-CoV-2) included brain fog, headache, numbness (tingling), dysgeusia, anosmia, myalgia, dizziness, pain that was not localized in the chest, blurred vision, tinnitus, movement disorder, focal motor and sensory deficit, dysarthria, ataxia, seizure, dysphagia, and aphasia [9].

Huang et al. conducted the largest and longest post-COVID-19 patient follow-up cohort analysis to date, demonstrating the importance of post-discharge care and longer follow-up [12]. After six months of discharge, most of the patients still showed at least one prolonged symptom, some of which were neurological or psychological complaints, whereas patients with a history of severe COVID-19 had a high risk of pulmonary diffusion abnormality. In addition, neutralizing antibodies at follow-up were also significantly lower than during the acute phase [12]. These findings raise a newfound urgency in the management of COVID-19 as it is now known that COVID-19 can have a variety of long-term effects, including one of the most concerning and increasingly visible long-term consequences of COVID-19: its effect on cognitive function, even in those with mild symptoms [6,8,13,14]. The World Health Organization recommended the importance of cognitive check-ups and rehabilitation for every COVID-19 patient who has recovered. However, intervention options that target cognitive dysfunction related to COVID-19 thus far are lacking and essential to be studied [15,16].

Citicoline, an essential substance for structural phospholipids of cell membranes, was found to have a neuroprotective effect on patients with neurological diseases [17,18,19,20,21]. Moreover, citicoline properties are also proposed to be able to limit inflammation and viral replication, leading to a cytokine storm, as happened in COVID-19 patients [17,22,23,24,25,26,27,28,29,30]. There is some evidence for the use of citicoline in neurological studies involving traumatic brain injury patients [31,32]. Citicoline was also found to be the only neuroprotective agent through confirmative clinical trials that has shown its safety and has a continuous beneficial effect on acute ischemic stroke in clinical stroke trials on improving post-stroke cognitive decline and functional recovery [33,34]. A prospective study also demonstrated the efficacy of citicoline against ischemic, hemorrhagic, and subarachnoid hemorrhage stroke in improving MMSE (Mini-Mental Score Examination) and Disability Rating Score [35].

We have to strongly consider that a meta-analysis and review by Martí-Carvajal et al. found that there is low-certainty evidence or even no difference between citicoline administration compared to the control group for the assessment of causes of death, disability, activity dependence, functional recovery, neurological function, and severe side effects [36]. Moreover, another Cochrane database systematic review by Fioravanti and Yanagi, based on data from the Specialized Register of the Cochrane Dementia and Cognitive Improvement Group, concluded that there is evidence of a positive effect of citicoline use on memory and behavior in patients with cognitive and behavioral disturbances in vascular cognitive impairment, vascular dementia, and senile dementia, although there was no evidence of a beneficial effect of CDP-choline on attention [37]. This review will cover the pathophysiology of COVID-19 and its neurological complications, along with the pharmacology of citicoline and its potential role in the management of COVID-19.

## 2. COVID-19 and Cognitive Decline

### 2.1. SARS-CoV-2 Infection Pathophysiology

Exploring the pathophysiology of SARS-CoV-2 infection is essential for identifying any feasible therapy options for COVID-19 patients among a plethora of non-specific treatment alternatives. Based on current information, SARS-CoV-2 is a positive single-stranded enveloped RNA virus that has spike (S) proteins on its surface with a high affinity for ACE2 receptors (Angiotensin Converting Enzyme 2) in humans. This virus uses ACE2 receptors for internalization and TMPRSS2 (Transmembrane Protease Serine 2) for priming the spike proteins [38,39]. ACE2 receptors are not only located in the lungs but also throughout various organs, including nerve tissue, blood vessels, gastrointestinal epithelium, and muscles, allowing SARS-CoV-2 infection to result in injury and even multi-organ failure [38,40,41].

The membrane fusion between SARS-CoV-2 and host cell plasma membrane is followed by an endocytosis process that regulates clathrin-independent and clathrin-dependent cell entry, and viral genomic material is released into the cytosol to be transcribed to the next RNA replication process [42,43]. The virus entry process will certainly be recognized as its antigen will be presented to the APC (antigen-presenting cell, e.g., dendritic cells or macrophages) [44].

Exposure to SARS-CoV-2 triggers MHC (HLA)-viral peptide restricted to T cells; T cell receptors recognize a single antigen binding unit on HLA molecules together with antigen peptides and the haplotype arrangement by HLA molecules alone was found to increase their binding capability to viral peptides on the surface of novel viral infections on the cell surface of APC (Antigen-Presenting Cells) [45,46]. The HLA system affects the incidence, clinical outcomes, and severity level of COVID-19, because the binding between HLA proteins and peptide epitopes significantly affects the immune response mechanism in patients [46,47,48,49]. Both the innate immune response and the humoral and cellular immune responses mediated by the adaptive T cell immune system play a role in combating SARS-CoV-2, but in the case of dysregulation of the immune response and excessive cell-mediated immune response, it will cause cytokine storm, namely excessive production of cytokines by immune cells such as dendritic cells (DCs), macrophages, natural killer (NK) cells, and adaptive T cells and B cells [50,51].

Cytokines and chemokines play an essential role as “messengers” in disease progression in patients infected with SARS-CoV-2, in addition to the findings of hyper induction of proinflammatory cytokines. These increase the rate of progression and mortality. In their use, cytokines and chemokines can be used as predictors of clinical symptoms and have diagnostic values, such as finding IP-10, IL-10, and IL-7 in asymptomatic COVID-19 patients that can help identify asymptomatic infections in close and suspected cases, as well as findings of IL-6, IL-7, IL-10, IL-18, G-CSF, M-CSF, MCP-1, MCP-3, IP-10, MIG, and MIP-1α are associated with COVID-19 severity [52,53]. Chemokine signatures in COVID-19 patients can vary according to their clinical symptoms, whether they are asymptomatic, mild to severely symptomatic, or post-infected [52].

### 2.2. SARS-CoV-2 Infection and Sirtuin 1 (SIRT1)

Sirtuin 1 (SIRT1 or silent mating type information regulation 2 proteins 1) is a pleiotropic protein able to target several transcription factors. It controls several intracellular pathways associated with cell death/survival [54,55]. SIRT1 is also crucial in the regulation of multiple interconnected networks for modulating dendritic and axonal growth. It has a protective effect on neuronal cells in terms of neuronal plasticity, cognitive function, and protection of neuronal degeneration in the prevention of age-related cognitive decline, which play a role in neurogenesis and gliogenesis [56]. SARS-CoV-2 infection was found to be associated with inhibition of SIRT1 activity [57]. Meta-analysis by Pinto et al. also showed that SIRT1 was up-regulated in the lung of patients with severe COVID-19 comorbidities [58]. SIRT1 has an anti-inflammatory function by inhibiting ADAM17 (A Disintegrin and Metalloproteinase Domain 17), also known as TACE (TNF-α converting enzyme) as well as other pro-inflammatory agents such as TNF-α, IL-6, and IL-1b; Therefore, in a condition where SIRT1 is decreased, causing inflammatory activity is not inhibited and hyperinflammatory response conditions such as in COVID-19 will not be controlled [59,60,61].

### 2.3. SARS-CoV-2 Infection and the Role of Phospholipase A2 Cascade

The role of phospholipase A2 (PLA2) in the inflammation cascade of COVID-19 has recently begun to be discussed [23]. A study by Abdalla et al. reported a positive correlation between plasma phospholipids depletion, the elevation of secretory phospholipase A2 (member of phospholipase A2 enzymes superfamily), and the occurrence of cytokine storms with the severity of COVID-19 [62]. Lipids indeed play a crucial role in the life cycle of the coronavirus. As in other viruses, the host lipids can be modulated and rearranged by the virus to reach a homeostasis condition optimum for its replication [63].

Concerning Abdalla et al.’s study, this result may occur because of phospholipid hydrolysis followed by the formation of lysophospholipids, which may, in turn, stimulate cytokine production [23]. PLA2 acts as a catalyzer of the arachidonic acid release, which generates pro-inflammatory lipid mediators such as prostaglandins, leukotrienes, lipoxins, and platelet-activating factors. Several sPLA2s, which among all the other phospholipase enzyme superfamily are proclaimed to be involved in many inflammatory conditions, induce exocytosis in macrophages, degranulate mast cells and eosinophils, and finally induce cytokine and chemokine production from macrophages, neutrophils, eosinophils, monocytes, and endothelial cells [64,65,66,67]. Free radicals will be produced in this cascade, leading to oxidative damage to the cell body [68]. Together, from all of these perspectives, the studies about the PLA2 enzyme bring great importance as it is a potential therapeutic cascade target that inhibits the production of any inflammatory lipid mediators and finally inhibits the cytokine storm.

### 2.4. SARS-CoV-2 Infection and the Role of Ubiquitin Protease System

Homeostasis of post-mitotic cells is controlled by the regulation of proteostasis, the equilibrium between protein synthesis, folding, intracellular trafficking, and degradation. This degradation is mostly handled through a major intracellular proteolytic pathway called the Ubiquitin Proteasome System (UPS) [69]. Inhibition of the UPS is known to be effective in reducing inflammatory response. It is also known that many viruses retain pro-viral or viral proteins in the process of antiviral proteins destabilization by manipulating the ubiquitination processes through the expression of their own deubiquitination proteins (DUBs) [25]. These mechanisms that include UPS’s vital role have also been described for SARS-CoV [26,70]. The UPS is important in the maintenance of cellular homeostasis and also in viral replication processes. Several studies have shown that virus infection leads to the accumulation of protein–ubiquitin conjugates. These mechanisms suggest an important role of the increased ubiquitination process in ubiquitin–proteasome-mediated viral replication or protein degradation. Later impact of the inhibition of proteasome activity causes a blockage of protein synthesis, endoplasmatic reticulum stress, and cell death, leading to the inhibition of viral replication [24].

### 2.5. SARS-CoV-2 Infection and Mitochondrial Dysfunction

Recently, SARS-CoV-2 has been found to create a genomic viral–mitochondrial interaction by way of viral genome integration into the host mitochondrial matrix and nucleus during cellular take-over in the transcription process. This causes impairment of mitochondrial function in energy metabolism and affects cellular oxygen utilization, initiating pro-inflammatory conditions as a reaction to hypoxic environment response [71,72,73,74,75]. Mitochondrial dysfunction can be the basis of neurological disorders in COVID-19 because neural networks are dependent on high oxygen levels to carry out their work. Additionally, the neurological manifestations of COVID-19, such as “brain fog” and other psychiatric manifestations, can be explained by the accumulation of neuronal dysfunction and mitochondrial metabolism, because cognitive work necessitates a continuous supply of oxygen at highly physiologic levels [71,76,77,78]. A high viral load in COVID-19 patients experiencing CNS involvements results in the compromise of neurons with high-level energy metabolism. Hypoxic conditions in brain tissue due to mitochondrial disorders are one of the underlying conditions of cognitive impairment, reducing protection toward infection and benefits the viral spreads [71]. Therefore, we propose that selective neuronal mitochondrial targeting in SARS-CoV-2 infection affects cognitive processes to induce ‘brain fog’ and results in behavioral changes that favor viral propagation.

### 2.6. SARS-CoV-2 Infection and Neurologic Complications

SARS-CoV-2, as part of the human coronavirus, has a neurotrophic potential similar to infection of SARS-CoV and MERS-CoV (Middle East Respiratory Syndrome Coronavirus), with invasion mechanisms of the central nervous system through two principles currently put forward: (1) Through the blood–brain barrier impaired permeability due to hyperinflammatory endothelial cells, as previously described; and (2) direct infection through peripheral nerves, olfactory sensory neurons, and then by axonal transport to the central nervous system [13,79,80]. The first report on detailed neurologic manifestations of COVID-19 patients in Wuhan by Mao et al. stated that neurologic symptoms were observed in more than one-third (36.4%) of the patients. These complications vary from dizziness, headache, impaired consciousness, acute cerebrovascular disease, ataxia seizure, and peripheral nervous system dysfunction such as impairment of taste, smell, vision, and nerve pain [7].

COVID-19 was soon realized to have a variety of significant long-term effects in addition to acute fatalities. Goertz et al. found persistent neurologic manifestations of muscle pain, dizziness, headache, weakness, and anosmia that continued up to 3 months from the onset of post-infection symptoms in 2113 patients in the Netherlands and Belgium [81]. Accumulatively, COVID-19 infection has been confirmed by numerous studies to cause many neurologic complications that vary from central neurological complications such as headaches, impaired consciousness, encephalopathy, delirium, seizures, cerebrovascular events, encephalitis, and autoimmunity, or peripheral neurological disorders such as cranial nerve dysfunction, and incidence of Guillain–Barre syndrome [7,8,9,16,53,82,83,84,85,86,87,88,89,90,91,92,93,94].

Endothelial dysfunction caused by COVID-19’s hyperinflammatory condition can damage neurovascular units in various end organs, including the brain [95,96,97]. One of the mechanisms is driven by neutrophil cells, monocytes, and macrophages, which play a major role in innate cellular immune response, found to accumulate at the SARS-CoV-2 virus port of entry site, which then triggers the addition of the pro-inflammatory cytokine IL-6 and TNF-α causes endothelial dysfunction and disrupts the blood–brain barrier. As a result, innate immune cells are carried into the brain and lead to the overactivation of the pro-inflammatory cytokine cascade, triggering thrombosis and inflammation [98,99].

Mao et al. reported that the same principle applies to how COVID-19 can impair peripheral nervous systems such as the olfactory organ [7]. A single-cell RNA sequencing gene expression analysis of human nasal biopsy samples study from Brann et al. reported that the supporting cells (particularly sustentacular and horizontal basal cells) and vascular cells (predominantly pericytes and immune cells of the macrophage/monocyte lineage) express both ACE2 receptors and TMPRSS2, which accommodate SARS-CoV-2 virus entry. After SARS-CoV-2 entry, local infection of support and vascular cells could cause significant inflammatory responses (including cytokine release) whose downstream effects could block effective odor conduction or alter olfactory sensory function neurons, leading to anosmia [100]. This may simultaneously interfere with the central nervous system and systemic viral spread, considering that the olfactory bulb is the only part of the central nervous system which is not protected by the dura mater. Retrograde or axonal transport from the nasal mucosa to the brain has been recently hypothesized for SARS-CoV-2. Once in the brain, it can disseminate to other regions of the brain, including the cortex and the hippocampus [79,101].

PCNS is worse in COVID-19 patients with comorbid chronic metabolic diseases (hypertension, diabetes mellitus, and obesity), cardiovascular disease, chronic lung disease, or a stroke history, who are more susceptible to SARS-CoV-2 infection, and who have a higher likelihood to experience organ dysfunction. Endothelial and blood–brain barrier disorders due to disruption of the RAAS (renin–angiotensin–aldosterone system) lead to a worse prognosis in the form of increased mortality or ICU admissions. This mechanism explains various neurological disorders that can occur due to SARS-CoV-2 infection, either during the acute infection period or prolonged after infection [102,103].

### 2.7. Cognitive and Functional Decline in SARS-CoV-2 Infection

The long-term effect of COVID-19 infection on the incidence of cognitive and functional decline remains unknown because the pandemic has only been around for roughly 2 years; but there has been an increasingly apparent and worrisome long-term complication on patients’ cognitive function, even in those with mild symptoms [7,13,14,96,104]. The most recent study by Hampshire et al. which analyzed a large volume of data from 81,337 individuals who recovered from COVID-19 showed that cognitive deficits persist even in the recovery phase. This cognitive deficit varied in scale with respiratory symptom severity, remained in patients with no other residual symptoms, was a greater scale than pre-existing cognitive problems, and could not be explained by differences in demographic or socioeconomic variables. The results also accord with other reports in demonstrating that ‘brain fog’, which implies trouble concentrating and difficulty finding correct words, is common in patients recovered from COVID-19 [105].

In a cohort study of 57 COVID-19 patients, Jaywant et al. found that, at a mean of 43.2 (SD = 192) days after initial admission, 46 patients (81%) had cognitive impairment (varied from mild to severe), with working memory deficits dominating cognitive domain dysfunction in 55% of patients, followed by set-shifting 47%, divided attention 46%, and processing speed 40%. This study took place with a population sample of 75% males, 61% non-white, mean age 64.5 (SD  =  13.9), 84% of them were previously living at home independently, 2 of 57 patients had preexisting cognitive dysfunction, and none had known dementia. With regard to COVID-19 severity, 88% of the patients had hypoxemic respiratory failure and 77% required intubation [16]. Another study by Zhou et al. reported cognitive dysfunction in the sustained attention domain, as evidenced by a continuous performance test, in 29 patients aged 30 to 64 who had recovered from COVID-19. Interestingly, this cognitive impairment was positively correlated with the degree of the inflammation, as objectively shown by c-reactive protein levels [93].

As many as 59–65% of COVID-19 patients in follow-up 3–4 months after hospital discharge had severe cognitive impairment, with the most affected impairments being on verbal learning and executive function, in addition to working memory, verbal fluency, and processing speed. Further investigations found that cognitive impairments were associated with the degree of pulmonary dysfunction and d-dimer levels during acute illness, suggesting restriction of oxygen delivery to the brain. However, there were no significant associations between cognitive status and the severity of COVID-19 status experienced by patients previously, which was measured by the length of hospitalization, total oxygen requirement and need of high-flow nasal cannula (HFNC), or severity markers such as lymphocytes, C-reactive protein (CRP), ferritin, or highest recorded d-dimer level, although higher d-dimer correlates with delayed verbal recall and psychomotor speed [8]. A survey study with a larger population of more than 1500 respondents who previously suffered from COVID-19 also revealed that memory impairment and difficulty concentrating were the ninth and the fourth most reported long-term symptoms after COVID-19, respectively [94].

A pattern of cognitive deficits was found among severe COVID-19 patients requiring intubation and mechanical ventilation in the ICU using MoCA (The Montreal Cognitive Assessment) scoring test. Patients with normal MoCA scores tended to show lower executive performance, while patients with mild to severe MoCA score deficits showed extensive cognitive impairment involving executive, memory, attention, and visuospatial functions. However, orientation and language were more inclined to be preserved [106]. In patients with ARDS (Acute Respiratory Distress Syndrome) who returned from hospitalization, based on cognitive profile testing, mood disorders, and anxiety using FAB (Frontal Assessment Battery) scoring, a cognitive deficit predominantly affected attention, mental processing speed, memory, and executive functions accompanied by a high prevalence of depression and anxiety [106,107,108]. One-third of COVID-19 patients are reported to have neurological symptoms and 20% of hospital-prone patients reported delirium symptoms of paranoid hallucinations, confusion, and agitation [7,96].

The pattern of cognitive deficits examined correlates with the degree of severity of COVID-19 patients. Patients in ICU care who develop delirium tend to have a poorer prognosis of cognitive function. However, cognitive impairment in patients with severe COVID-19 and ARDS due to other diseases does not correlate with the length of time on mechanical ventilation and time spent in the ICU. Incidence of stroke or impaired perfusion must also be considered among COVID-19 patients who experience cognitive dysfunction as coagulopathy and thrombosis are often reported in many COVID-19 patients [106].

Cognitive decline in COVID-19 patients is caused by inflammation of multisystemic organs that have intertwining roles with each other, in the form of: (1) Hypoxemia due to pulmonary organ damage indirectly causing nerve damage can proceed to cognitive decline [6]. The characteristic feature of COVID-19 patients is that they can experience silent hypoxemia, wherein oxygen levels drop below normal levels but do not cause dyspneic symptoms, which then delays the treatment of hypoxemia [76,109,110]; (2) thrombosis and coagulopathy due to systemic vasculitis, hyperinflammatory conditions that lead to a cytokine storm can cause multi-organ damage, as well as susceptibility to silent infarcts due to microemboli. This condition is found in COVID-19 patients with severe symptoms and shows an increased risk of developing delirium, which can progress to cognitive decline [111,112]; (3) damage and dysfunction of the blood–brain barrier due to pro-inflammatory cytokines, which interfere with its permeability, causing microglial activation and oxidative stress. The result is neuroinflammation, with a neuronal injury that can progress to delirium in the short term and cognitive deficits in the long term [113,114]. All of these mechanisms contribute to the development of cognitive deficits, particularly impairing PLA2 and autophagy activity, both of which have been linked to cognitive impairment in neurodegenerative diseases such as Alzheimer’s disease [30,115,116,117,118,119,120,121,122].

Human coronavirus infection in the central nervous system was observed to focus on the temporal area, especially on the hippocampus as a function of learning. Furthermore, spatial orientation was found to be more susceptible to neuronal cell necrosis and apoptosis in the CA1 and CA3 subregions in an in vitro study using intracerebral inoculation of the HCoV strain OC43 [123,124]. CA3 itself plays a role in episodic memory with multi-element recollection holistically, including memory recall and mnemonic pattern creation for memorization [124]. The hippocampus, which is the part of the brain that plays an essential role in working memory, episodic memory, and spatial learning, tends to develop memory deficits if damaged or degenerated. The hippocampus, which is involved in memory formation, is especially susceptible to respiratory viral infections, as shown in animal models [123]. Short-term deterioration in hippocampus-dependent learning and reduced long-term potentiation is associated with the influenza virus [125]. The incidence of Alzheimer’s dementia itself is closely related to a linear relationship between cognitive decline and hyperactivity of neural networks in the hippocampus, medial temporal lobe, and parts of the cortex [13,126]. Therefore, the presence of nerve damage due to SARS-CoV-2 infection in the central nervous system can explain the relationship of cognitive impairment in patients after SARS-CoV-2 infection, increasing the risk of developing Alzheimer’s dementia.

The World Health Organization has recommended that every COVID-19 patient who has recovered undergo a cognitive check and rehabilitation. However, intervention options that target cognitive dysfunction related to COVID-19 at present are lacking and are essential to be studied [15,16]. Barriers to data collection and cognitive complication-related trials in COVID-19 patients were also due to the prioritization of symptoms and other emergency interventions, lack of time, and the overwhelmed healthcare system in this pandemic situation [127]. Nevertheless, lessons learned from the consequences of previous epidemics caused by coronaviruses with JP Rogers et al.’s study revealed a decrease in cognitive function for a period from 6 weeks to 39 months in more than 18% of patients who had MERS-CoV or SARS-CoV [128].

Early detection of neuropsychological symptoms in COVID-19 patients is important to prevent the risk of modifiable irreversible disorders and prevent further cognitive decline. Cognitive decline is often diagnosed at an advanced stage accompanied by functional disruption. Cognitive decline does not occur independently but rather in a series of quality-of-life decrements that affect ADLs (activities of daily living) and IADLs (instrumental ADLs) abilities [6]. Almeria et al. in a cohort study of neuropsychological assessment of COVID-19 patients with no history of age-related cognitive impairment or CNS and psychiatric disease found lower working memory scores in patients with symptoms of headache, anosmia, and dysgeusia, and also a decrease in coding memory assessments, attention, complex memory work, data processing speed, executive function, denomination, and the global cognitive index. Decreased working memory in several areas such as complex memory, visual memory, verbal memory, attention, and executive functions were also found in patients with diarrheal symptoms, patients who needed oxygen therapy, and patients in ICU care [129].

In patients with pulmonary disease, central nervous system infections, cerebral hypoxia, and chronic hypoxia are correlated to temporary or permanent cognitive deficits indicated by decreased daily activities due to impaired attention, memory, and executive function [129]. Patients over 65 years of age—a demographic with neurocognitive frailty—often have a history of mild cognitive impairment (MCI), which puts them at a higher risk of developing severe manifestations of COVID-19 such as delirium and later cognitive deficits. However, delirium and cognitive deficits are often overlooked or underdiagnosed. COVID-19 inpatients are often admitted to isolation rooms where contact with health workers or family visits are minimal, especially patients with long-term sedation or ventilation in the ICU, which sometimes complicates assessment and treatment due to severe agitation related to their delirium [6].

Mental health has also become an issue, especially for elderly COVID-19 patients. These issues include feelings of abandonment, isolation, and neglect due to isolation. Elderly patients with cognitive impairment and dementia are prone to experience feelings of worry, anger, depression, agitation, and isolation when facing the community stigma towards COVID-19 infected patients [130]. The association between cognitive impairments and mental issues such as anxiety or depression is bi-directional; a more severe cognitive deficit may bring more anxiety and depression due to a higher need of overcoming cognitive challenges, and conversely, more severe anxiety and depression may impair cognitive performance more [8]. Emotional and social support are much needed. However, we know that there are limitations in visiting isolated patients, both by family and health workers; therefore, emotional and social support must be addressed using other creative methods such as utilizing technology and promoting engagement via social media interactions.

### 2.8. Properties of Citicoline toward Cognitive Function

To date, no “anti-COVID-19” treatment has been shown to be the most potent and curative. Pharmacological treatments of COVID-19 patients are still “potential” treatments that treat symptomatically, prevent progression (e.g., anti-inflammatory corticosteroids), regulate the body’s immune response (e.g., immunosuppressant toxilizumab or antibody-based therapy using convalescent plasma therapy), or are drugs with antiviral pathway mechanisms, such as the anti-retrovirus lopinavir (LPV), the adenosine analog antiviral remdesivir (GS-5734), the selective influenza virus synthetic pro-drug inhibitor favipiravir (FVP), the broad-spectrum antiviral/anti-parasitic nitazoxanide, or even the use of the antihelmintic ivermectin and the antimalarials chloroquine and hydroxychloroquine [42]. However, there are still no potential clinical trials on treatment approaches for cognitive and neurological symptoms in patients with acute COVID-19 infection or patients with prolonged symptoms. For this reason, the potential of citicoline is discussed in this study, along with a comprehensive review of its pathophysiological approaches.

### 2.9. Profile of Citicoline

Cytidine-5’-diphosphocholine (CDP-choline), also known generically as citicoline in an international non-proprietary designation, is a chemical substance that is identical to the natural metabolite phospholipid phosphatidylcholine precursor that works on intracellular phospholipids synthesis and is also an exogenous source for choline and cytidine [17,131]. Citicoline has favorable side effects and low toxicity levels in the human body [21,132]. Citicoline, since the 1900s, has already been proven to increase cerebral blood flow velocity and reduce the pulsatile and resistance indexes by enhancing cerebrovascular perfusion in DA patients. It also significantly increases brain bioelectrical activity, improves cognitive performance, and seems to be potentially valuable in patients with the apolipoprotein E ε4 allele risk factor [133,134]. Today, it is known in many countries as a drug and as a dietary supplement globally [135].

### 2.10. Citicoline Acts on Protective Mechanism by Increasing SIRT1 Expression

Sirtuin 1 (SIRT1 or silent mating type information regulation 2 proteins 1), encoded by the SIRT1 gene, is a protein that is crucial in the regulation of multiple interconnected networks for modulating dendritic and axonal growth. It has a protective effect on neuronal cells in terms of neuronal plasticity, cognitive function, and protection of neuronal degeneration in the prevention of age-related cognitive decline, which plays a role in neurogenesis and gliogenesis [56]. SARS-CoV-2 infection was found to be associated with inhibition of SIRT1 activity [57]. The problem with this impaired mechanism is SIRT1 protein is known to have a role in alleviating the oxidative stress that is significantly increased in neurodegeneration. This significant decrease in SIRT1 will disrupt cellular functionality due to the accumulation of oxidative stress, mitochondrial damage, and neuroinflammation [136].

On the other hand, the tumor protein p53 had an increased expression along with an increase in blood concentrations of proinflammatory cytokines [137,138]. The p53 is an intracellular defense mechanism against viruses, a key gatekeeper for cellular division and survival that regulates innate immune response, which is also a target for SARS-CoV-2 to deploy its mechanism [139]. Increased activation of p53 will increase the incidence of apoptosis both in cells infected with SARS-CoV-2 and in normal cells, so that it can cause tissue injury [140]. These conditions of increased p53 expression suggest a dysregulation of the p53/SIRT1 axis in COVID-19 patients leading to uncontrolled regulation of inflammation [138]. Disruption of SIRT1 expression will disrupt cellular functionality due to the accumulation of oxidative stress, mitochondrial damage, and neuroinflammation that end in neurodegenerative diseases, one of which is also related to Alzheimer’s disease; impaired SIRT1 expression will cause neuronal inflammation and accumulation of Aβ plaques [136,141,142,143].

The neuroprotective action of citicoline in an in vivo cerebral ischemic stroke study was found to up-regulate the expression of SIRT1 protein levels in the brain, cultured neurons, and circulating blood mononuclear cells, although citicoline was also found to be unable to reduce infarct volume in the brain [131,144]. SIRT1 has an anti-inflammatory function by inhibiting ADAM17 (A Disintegrin and Metalloproteinase Domain 17), also known as TACE (TNF-α converting enzyme) as well as other pro-inflammatory agents such as TNF-α, IL-6, and IL-1b; Therefore, in a condition where SIRT1 is decreased, inflammatory activity is not inhibited and hyperinflammatory response conditions such as in COVID-19 will not be controlled [59,60,61]. Citicoline acts as a SIRT1 activator, and by increasing its expression, concomitantly induces the neuroprotective properties of SIRT1 and beneficially in cognitive reservation and prevention of other neurologic disorders in COVID-19 [145].

### 2.11. The Anti-Inflammatory Actions of Citicoline on Phospholipase A2 and Mitochondrial Dysfunction

The human brain contains a large number of lipids, composed mostly of phospholipids. As the major structural part of cell membranes, phospholipids serve to maintain the optimal function of cell membranes. In neuronal membranes, it facilitates the conduction of nerve impulses and neurotransmission. Neural growth and regeneration impairments in the CNS have been involved with the impaired metabolism of phospholipids, and this condition is also associated with neurodegenerative disease and neuroplasticity [19,146]. Membranes in patients with Alzheimer’s Disease (AD) are significantly lower than in non-cognitive impaired patients. As shown by post-mortem brain analysis, there is a decrease in choline levels among patients with AD [120,121,122].

Wang et al. found that utilizing citicoline, they were able to increase phospholipid metabolism by considerably boosting phospholipid levels in PC12 cells and rat brain in vitro. PC12 cells are the cell lines from the pheochromocytoma of rat adrenal medulla, which has been commonly used in research related to neurotoxicity, neuroprotection, and neurosecretion as PC12 cells characteristically secrete catecholamines, dopamine, and norepinephrine. Moreover, PC12 cells also have ion channels and neurotransmitter receptors [147,148]. As in the case of cerebral ischemia or when choline levels are depleted, phospholipids will be hydrolyzed by phospholipase A2 (PLA2) which can impact arachidonic acid release. This release will also form reactive oxygen species, lipid peroxides, and toxic aldehydes (malondialdehyde, 4-hydroxynonenal, and acrolein), which damages CNS. Although citicoline is not a “direct PLA2 inhibitor,” as an intermediate in phosphatidylcholine synthesis, it can be used to minimize phospholipid hydrolysis, attenuate the increase of PLA2 activity in both the membrane and mitochondrial fractions, and correct age-related changes within the brain neuronal membrane [23,30]. Citicoline has the potential to repair mitochondrial malfunction, which is one of the contributors of neurological problems in COVID-19, by retaining sphingomyelin and cardiolipin; an exclusive inner mitochondrial membrane component [71,76,77,78,149,150]. These potential mechanisms of citicoline may explain its beneficial effects towards several brain diseases, including stroke, Parkinson’s disease, and Alzheimer’s disease [149,151,152,153].

### 2.12. The Anti-Inflammatory and Anti-Viral Actions of Citicoline on Ubiquitin Proteasome System

As degradation of proteostasis regulation is mostly handled through a major intracellular proteolytic pathway called the Ubiquitin Proteasome System (UPS) and as many viruses, including SARS-CoV, retain pro-viral or viral proteins by manipulating the ubiquitination processes through the expression of their own deubiquitination proteins (DUBs), citicoline shows potential to have an anti-inflammation plus anti-viral effect on the UPS mechanism since it functions as proteasome regulator by shifting the population in favor of capped proteasome particles, activates the intrinsic activity of the 20S and 19S to form the 26s particle, and it plays a modulatory role through a fine tuning between activation and limitation of its activity [24,25,26,69,70,154,155,156]. Several studies have shown that virus infection, including SARS-CoV, leads to the accumulation of protein–ubiquitin conjugates. These mechanisms suggest an important role of the increased ubiquitination process in ubiquitin–proteasome-mediated viral replication or protein degradation. The after impact of the inhibition of proteasome activity causes a blockage of protein synthesis, endoplasmatic reticulum stress, and cell death, leading to the inhibition of viral replication [24].

The UPS is known to be important for virus endocytosis and maturation process. Proteasome inhibitors (lactacistin and MG132) have an inhibitory effect which is involved in the early stages of virus replication. Although it does not block the internalization of the virus, it makes the virus remain in the vesicles, both endosomes and lysosomes, therefore virus cannot be released into the cytosol [69]. Another study of UPS inhibition in an airway smooth muscle cell line by Moutzouris et al. also suggested that when UPS is inhibited, levels of IL-6, sICAM-1, IP-10, MCP-1, MIF, and RANTES decrease. It also impacts in the up-regulation of MKP-1, a negative regulator of the serine/threonine protein kinases MAPK, which plays a vital role in proliferation to migration and synthesis of fibrotic and inflammatory proteins, including cytokines [157].

### 2.13. Effects of Citicoline on the Neurotransmitter System

Acetylcholine is an essential neurotransmitter that is sensitive to metabolic and other changes. Deficiency in cholinergic transmissions is hypothesized to be the cause of delirium, one of the most common and severe neurological complications in COVID-19 patients [86,87,88,158,159,160]. This hypothesis was first proposed after it was observed that delirium occurs when poisons and medications that inhibit cholinergic function were consumed [161]. Thus, as a CDP-choline exogenous donor, citicoline is hypothesized to play a key role to manage delirium by enhancing cholinergic activity [162]. A randomized control trial of 81 patients undergoing hip fracture surgery (HFS) showed that delirium incidence was lower in the group treated with citicoline (11.76%), compared to the placebo group (17.39%). However, the findings were insufficient to draw any conclusion [163]. When compared to a meta-analysis that looked into the use of cholinergic enhancers in elderly patients undergoing HFS, the results showed that using cholinergic enhancers could reduce the risk of postoperative delirium, though large-scale randomized controlled trials are needed to confirm these findings [159].

The cholinergic system including choline acetyltransferase (ChAT) and acetylcholinesterase (AChE) is essential in carrying out cognitive functions, as evidenced by the decrease in neurotransmitter signaling in line with the progression of cognitive impairment that occurs in various neurodegenerative diseases such as Alzheimer’s disease, even though the systems affected are usually complex and involve several pathways [164,165]. However, the discovery that neuronal plasticity response can increase this cholinergic system activity suggests a neurorestorative mechanism in the early stages of cognitive impairment, implying that citicoline could be a potential drug that enhances synaptic neurotransmitter activity to improve or prevent worsening of cognitive function [135,165,166,167]. Cholinergic transmission refers to acetylcholine transmitters that respond to two types of receptors: (1) muscarinic acetylcholine receptors (mAChR) including subtypes M1, M2, M3, M4, and M5 that is distributed in the CNS; and (2) ionotropic nicotinic acetylcholine receptors (nAChR) including subunits α, β, δ, and γ [164]. The hippocampus, brain cortex, and thalamus are highly dense with mAChR and bind transduced acetylcholine signals in the CNS, controlling the release of neurotransmitters and cognitive function in learning and memory. In contrast, nAChR controls the release of neurotransmitters from presynaptic sites [168]. In a study of the cholinergic system during AD progression through measurement of mRNA expression levels in nucleus basalis neurons, Mufson et al. found no changes of mAChR subtypes M1–M5, nAChR subunits α1–6, and β1–4 in patients with AD or MCI compared to the group with no cognitive impairment (NCI). Simultaneously, there was a significant up-regulation of nAChR subtype α7 expression in CBF neurons in the AD group compared to the NCI and MCI groups that is inversely related to cognitive performance and suggests cellular degeneration [164].

Citicoline acts as exogenous CDP-choline after hydrolysis. It is absorbed as cytidine and choline and would be re-synthesized by cytidine triphosphate phosphocholine-cytidylyltransferase in the brain. Moreover, choline has a role in the neurochemical process as a precursor and metabolite of acetylcholine [169,170]. The vital cholinergic system can be enhanced by citicoline. Choline can be an additional external substrate resource for the synthesis of acetylcholine, which is essential in cognitive performance. Additionally, citicoline may also boost other neurotransmitter levels in synapses by increasing dopamine, serotonin, and norepinephrine, all of which are thought to have neuroprotective properties [135,154]. In AD, it has been shown that the combination of citicoline as adjunctive therapy with cholinesterase inhibitors (AChEIs) shows improvement in cognitive, mood, and behavioral symptoms than AChEIs treatment without citicoline [167].

### 2.14. Other Citicoline Anti-Inflammatory, Neuroprotective, and Neurorestorative Properties

Citicoline also has other neuroprotective activities as it affects cell energy balance, glutamate excitotoxicity, oxidative cascade, apoptosis, and endothelial barrier disruption [171]. Firstly, concerning cell energy balance, citicoline corrects cell energy deficiency and restores neuronal ionic balance by stimulating Na+/K+ ATPase activity, as it can also restore membrane integrity by preventing loss of neuronal ATP levels [154,172,173]. Secondly, citicoline decreases neuronal glutamate efflux by delaying the reversal of neuronal glutamate transporters and increasing astrocytes glutamate uptake by increasing excitatory amino acid transporter 2 [154,173,174]. Third, in the oxidative cascade, citicoline can prevent PLA2 activation as previously explained, stimulate glutathione synthesis by inducting glutathione reductase activity, and attenuate lipid peroxidation [149,154,175,176,177]. Fourth, by increasing BCL-2 expression together with Silent Information Regulator 1 (SIRT1), citicoline can play a role in anti-apoptosis by decreasing procaspase and caspase expression [144,178,179,180]. Last but not least, citicoline can affect endothelial barrier disruption by regulating tight junction proteins, which reduces brain edema [181]. Along with its neuroprotective effects on the nervous system, citicoline was also found to have a positive impact on neuroregeneration by maintaining the function of neurogenesis, synaptogenesis, gliogenesis, angiogenesis, and preserving the structure of neuronal morphology [171].

Citicoline, which has been more known for its potential as an add-on therapy in neurodegenerative, neurovascular, and traumatic brain disorders due to its neuroprotective and regenerative characteristics, has also been investigated for its potential in neuroinflammatory diseases. It was observed that CDP-choline and choline decrease cytokines/chemokines MIP-1α, TNFα, IL-1β, and MCP-1 secretions, and the second component of CDP-choline, cytidine, specifically decreases IL-6, RANTES, and anti-inflammatory cytokine IL-10 secretions [182]. From this study, it can be stated that citicoline has the potential properties for neural anti-inflammation in addition to its more known neuroprotective and regenerative properties.

### 2.15. The Potential Roles of Citicoline in COVID-19-Related Cognitive Decline and Other Neurologic Complications

**Figure 1:**
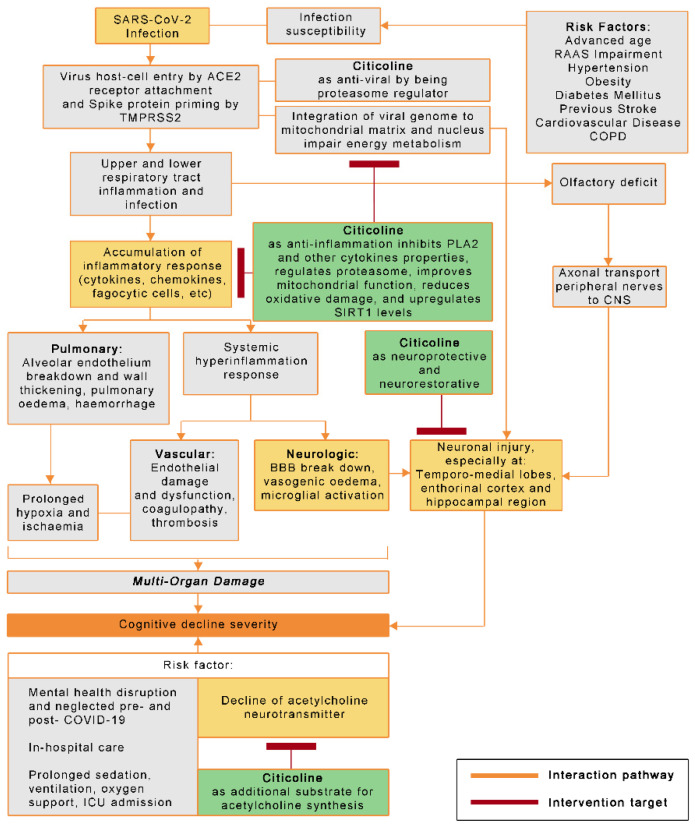
The role of citicoline in COVID-19 pathophysiology towards cognitive and other neurologic complications. Notes: ACE2 receptor (Angiotensin Converting Enzyme 2 receptor); BBB (Blood–Brain Barrier); CNS (Central Nervous System); COPD (Chronic Obstructive Pulmonary Disease); PLA2 (Phospholipase 2); RAAS (Renin-Angiotensin-Aldosterone-System); TMPRSS2 (Transmembrane Serine Protease 2).

Citicoline has an influential role that could potentially serve as adjunctive therapy and prevent COVID-19 related cognitive decline and other neurologic complications. Citicoline plays a role (Figure 1) in anti-inflammation, anti-viral, neuroprotection, and increases the neurotransmitter synthesis of acetylcholine.

With regard to citicoline’s anti-inflammatory and neuroprotective effects, recent studies have found the correlation between the role of phospholipase A2 in the COVID-19 inflammation cascade and the occurrence of cytokine storms [23,62]. Citicoline can attenuate the increase in PLA2 activity by being a source of choline to prevent hydrolysis of phospholipids when internal choline levels are depleted. It also helps to stimulate the repair and regeneration of damaged mitochondria membranes by preserving sphingomyelin and cardiolipin levels [149,175,176,177,183,184]. Citicoline also shows both potential to be anti-inflammation plus anti-viral on the UPS mechanism since it functions as a proteasome regulator by shifting the population in favor of capped proteasome particles, activates the intrinsic activity of the 20S and 19S to form the 26s particle, and it plays a modulatory role through a fine tuning between activation and limitation of its activity [24,25,26,69,70,154,155,156]. Citicoline was also found to be capable of triggering SIRT1 expression up-regulation, which through modulating dendritic and axonal growth, is beneficial for neuronal plasticity and cognitive function. Up-regulation of SIRT1 expression also reduces the neuronal inflammatory response in hyperinflammatory conditions such as SARS-CoV-2 infection, aiding in neuroprotection and restoration [57,59,60,61,138].

Citicoline may also have an advantage in overcoming the mitochondrial dysfunction known to occur in COVID-19, which underlies neuronal dysfunction and cognitive impairment due to impaired energy metabolism and tissue oxygen supply [71,185,186]. This is based on in vivo studies that show that citicoline reduces tissue damage caused by ischemia and reperfusion by improving mitochondrial function and reducing oxidative damage. Citicoline also has a mitoprotective effect and improves mitochondrial functional status in the neocortex by (1) preventing mitochondrial cyclosporin-A-sensitive pores from opening and (2) restoring mitochondrial transmembrane potential, both of which have been shown to have benefits to increase cognitive processing [185,186]. Citicoline was also found to prevent increased mitochondrial PLA2 activity, thereby preventing energy failure in ischemic brain conditions [187].

As the prevalence of neurological complications due to COVID-19 is relatively higher in more severe patients and an increase in cytokines correlates to a worse clinical prognosis, the use of citicoline may also be advantageous as it decreases the cytokines MIP-1α, TNFα, IL-1β, MCP-1 secretions, IL-6, RANTES, and the anti-inflammatory cytokine IL-10 secretions [182,188]. Citicoline also has other neuroprotective properties as it affects cell energy balance, glutamate excitotoxicity, the oxidative cascade, apoptosis, and endothelial barrier disruption. Citicoline was also found to have many positive effects on neuroregenerative mechanisms by maintaining the function of neurogenesis, synaptogenesis, gliogenesis, angiogenesis, and preserving the structure of neuronal morphology [144,149,171,172,173,174,175,176,177,178,179,180,181].

In addition to its potential effects towards anti-inflammation along with its neuroprotective and neurorestorative properties toward COVID-19, citicoline has been known to positively affect neuronal network capability in cognitive domains ranging from working memory, episodic memory, empirical method, attention, to processing of spatial memory; and it also influences cellular physiology on hippocampal and brain cortex neurons [189]. AD patients treated with citicoline and acetylcholinesterase inhibitors have shown statistically significant improvements in cognitive scoring using MMSE [190]. Although citicoline has shown much potential, in a meta-analysis and review by Martí-Carvajal et al. in several randomized controlled trials (RCTs) regarding the use of citicoline orally, intravenously, or a combination of both in acute ischemic stroke patients, it was found that there is low-certainty evidence or even no difference between citicoline administration compared to the control group for the assessment of causes of death, disability, activity dependence, functional recovery, neurological function, and severe side effects [36]. Citicoline is frequently used in several European countries in the management of cognitive impairment as the dominant clinical feature in cerebrovascular diseases, although in its development, there have been changes in terms of dose, administration method, and criteria for therapeutic indications; and Fioravanti and Yanagi’s research based on data from the Specialized Register of the Cochrane Dementia and Cognitive Improvement Group concluded that there is evidence of a positive effect of citicoline use on memory and behavior in patients with cognitive and behavioral disturbances in vascular cognitive impairment, vascular dementia, and senile dementia, although there was no evidence of a beneficial effect of CDP-choline on attention [37]. Despite the potential use of citicoline in the treatment of acute stroke, there is still controversy about its effectiveness, and since 17 April 2009 the use of citicoline has been discontinued in the USA and Canada, due to the failure to use citicoline as a neuroprotective agent in terms of clinical benefits for the treatment of ischemic stroke [36,191].

## 3. Conclusions

With rising concerns about COVID-19 hyperinflammation and its potentially damaging effects on the neurovascular system, it is more necessary than ever to consider treatment options for managing short- and long-term effects on neurological complications, particularly cognitive function. While maintaining adequate phospholipid structure and function in brain cells, citicoline, which is identical to the natural metabolite phospholipid phosphatidylcholine precursor, could play a potential role in combating COVID-19-related cognitive decline and other neurologic complications through its anti-inflammation, anti-viral, neuroprotective, neurorestorative, and acetylcholine neurotransmitter synthesis properties. As there are still many both positive and negative arguments, we have to consider the result from recent citicoline Cochrane reviews. More evidence is needed involving randomized and observational studies in patients with COVID-19 and post-infected patients to evaluate the use of citicoline in reducing the incidence of cognitive impairment and other neurologic complications.
